# The Best Means to Preserve Leeches Healthy

**Published:** 1853-03

**Authors:** Gustavus Schuler


*The best Means to preserve Leeches Healthy.* By GUSTAVUS SCHULER.—The apothecaries in Moldavia are, according to the sanitary laws, compelled to keep constantly a stock of at least 300 healthy and serviceable leeches. But, particularly as the hot summer months are the most prejudicial season for these animals, it often happens that in the time of the greatest need no leeches are to be obtained.

Amongst the various means which have been recommended to keep leeches healthy, and to restore the sick ones, good well-burnt wood charcoal has proved, according to my experience, to be the best, as shown by the following experiment. I washed the charcoal well three or four times with fresh spring water, to separate the adhering ashes, and then laid it, while wet, and without breaking it smaller, in a large glass cylinder; put the sick leeches, recently washed, into this cylinder, but did not give them any more water, as enough was to be found in the washed charcoal. The glass was tied over with a piece of linen and placed in a cellar, where for five days the leeches were resigned to their fate.

After this period I found the leeches, to my satisfaction, perfectly well. They were quite in a condition to be used, which was not the case in their sick condition.

For two years I have treated my leeches in this way, and always retained them healthy and serviceable. The number of deaths amongst them has been very small. The only precaution I observe is to place all my leeches for eight days in summer on recently washed charcoal, and for two weeks in winter. The method is a cheap one, and one easy of execution.—*Buchner's Repertorium, from Annals of Pharm. for Aug.* 1852.

